# Supplementary material for: Chromothripsis during telomere crisis is independent of NHEJ, and consistent with a replicative origin
Source: Genome Res. 2019 May;29(5):737–49. doi: 10.1101/gr.240705.118 (PMC6499312; doi:10.1101/gr.240705.118)
Supplement: Supplemental Material [file supp_gr.240705.118_Supplemental_file_1.zip › contigs/annotated_contigs/DB79/contig.2.DB79_length_552_mean_cov_7.0652173913.docx]

**DB79_length_552_mean_cov_7.0652173913**

```
CTAGAAACACATGGATATTCTCCACTTTTAAAAATTCCATATTGACCCCATGTTTCTCTCAGCTTTTGCCTTCCCTATCTCCTTCTCTT
 >chr7:141338902-141339191 - E=3e-159
CAAAGTCAGGTTTCTTGAAGGAGTAGCCTGTCCTTGCTGTCTCCACTTCTTTACATCACTTTTATTTCTTGACTTTCTTCAGTCTGGCT

TCAGCCACCACACTTTGGAGGCTGCCCAGCTTTTGGAGGCTGCCAAATTCGATGGATACTCTCTTGTCTGTATATTTAAAGTCTTTGCT

TTTGCTTCTCGAAACTCTTA|CT|AAAGTAGACCTCCAAAATCAGTTTTAGAGACTTAACTAGAGCAGGAGAAGCCTCTCAGGGATCAC
                     >chr7:141341251-141341516 + E=8e-148
TGAATTCATCCTTCTGTGTGCCACCAGAGCAGGCCCAAGCAATGTGCCATCAATGGGCATAGCAGTCTCTGTGCACAGTCTTTCTGTTA

ACGTGACTGCTTTTGGTAGTGATGTTTTAGGCACAGAGATGCCATTTTTACCTCTATGTATAAGAAGCATGAAATAAATAAAATGAAGG

CAGAGTCAGCAGAAAGGTTG
```
